# Effects of a multidomain intervention on physical performance and sarcopenia in long-term care facility residents: The I-COUNT pilot randomized controlled trial

**DOI:** 10.1016/j.jnha.2026.100943

**Published:** 2026-08-13

**Authors:** Chiara Ceolin, Giulia Pasolini, Mario Virgilio Papa, Giacomo Scotti, Alvise Bobbo, Eleonora Macchia, Alessia Ghidini, Zaira Romeo, Giuseppe Sergi, Marina De Rui, Simona Gentile, Stefania Maggi, Alessandro Morandi, Marianna Noale, for the I-COUNT study group

**Affiliations:** a Department of Medicine (DIMED), University of Padua, Padua, Italy; b Department of Neurobiology, Care Sciences and Society, Karolinska Institutet and Stockholm University, Aging Research Center, Stockholm, Sweden; c Department of Clinical and Experimental Science, University of Brescia, Italy; d Neuroscience Institute, National Research Council, Padova, Italy; e Azienda Speciale Cremona Solidale, Cremona, Italy; f Geriatrics Unit, University Hospital of Padua, Padua, Italy

**Keywords:** Long-term care facilities, Multidomain intervention, Physical performance, Muscle strength, Sarcopenia

## Abstract

**Background:**

Population aging requires multidimensional strategies to preserve physical function and prevent sarcopenia. The I-COUNT pilot study evaluated the effect of a multidomain intervention in long-term care facilities (LTCFs) residents, hypothesizing improvements in physical performance, muscle strength, and sarcopenia compared to usual care.

**Methods:**

Residents aged ≥70 years from two Italian LTCFs were screened, and 90 participants were enrolled in a 6-month pilot cluster-randomized controlled trial and allocated to a multidomain intervention (physical training, cognitive stimulation, Mediterranean diet enriched with functional foods, and vaccination promotion) or usual care. Physical performance (Short Physical Performance Battery, SPPB), handgrip strength, body composition, and sarcopenia according to European Working Group on Sarcopenia in Older People (EWGSOP2) criteria were assessed at baseline and after 6 months. Changes over time were analyzed using mixed-effects models adjusted for age, sex, and study center.

**Results:**

Baseline characteristics were comparable between groups. At 6 months, participants receiving the intervention showed significant improvements in physical performance (SPPB: +1.6 points vs. + 0.3 in controls; between-group difference: +1.8 points, *p* = 0.001), and muscle strength (handgrip: +3.2 kg vs. −0.1 kg in controls; between-group difference: +3.8 kg, *p* = 0.026). No significant between-group differences were found for the 6-minute walk test or anthropometric measures. Appendicular skeletal muscle mass (ASMM) tended to be preserved in the intervention group while declining in controls (between-group difference approaching +0.9 kg, *p* = 0.057). Sarcopenia prevalence decreased in the intervention group (37.1% to 15.4%) and remained stable in controls (28.4% to 29.9%), with a borderline group-by-time interaction (*p* = 0.109).

**Conclusions:**

A multidomain intervention is safe, feasible, and potentially effective for improving physical performance and preventing the progression of sarcopenia in LTCF residents. Given the pilot nature of this study, these findings warrant confirmation through larger randomized controlled trials.

**Clinical trial number:**

NCT06820710.

## Highlights

• 90 LTCF residents randomized to the 6-month multidomain intervention or usual care.
• I-COUNT combined exercise, cognitive training, diet, and vaccination promotion.
• SPPB improved +1.8 points more with intervention vs. usual care.
• Handgrip strength rose +3.8 kg more in intervention group.
• Sarcopenia prevalence fell from 37.1% to 15.4% with intervention.

## 1 Introduction

Institutionalized older adults are among the most vulnerable groups in the aging population, as they typically exhibit reduced intrinsic capacity across both physical and mental domains [1]. In this setting, frailty, defined as an age-related state of decreased physiological reserves associated with increased vulnerability to adverse outcomes [2], affects 30–70% of long-term care facility (LTCF) residents [3]. Frailty has consistently been associated with disability, falls, hospitalization, institutional dependency, and death [4]. Alongside frailty, sarcopenia represents another major geriatric syndrome contributing to adverse outcomes in older adults [5]. Frailty and sarcopenia share several pathophysiological mechanisms, including chronic low-grade inflammation, hormonal dysregulation, malnutrition, physical inactivity, and impaired neuromuscular function, and frequently coexist in the same individuals. The prevalence of sarcopenia among LTCF residents has been estimated at approximately 40% [6]. This high prevalence is likely related, at least in part, to the sedentary lifestyle and physical inactivity that commonly characterize institutionalized populations. Inactivity promotes a cascade of age-related physiological alterations, including loss of muscle mass, deterioration of neural mechanisms involved to strength and power production, reduction in bone mineral density, and cognitive decline [7].

Although the transition from frailty to robustness is uncommon, frailty is increasingly recognized as a dynamic process, providing opportunity for prevention and intervention [8]. Accordingly, strategies targeting vulnerable older adults should address multiple domains, including physical function, nutrition, and cognition [7]. Multicomponent exercise programs combining resistance training, balance exercises, and gait retraining have shown beneficial effects on frailty and physical function; however, evidence in LTCF residents remains limited [9].

The Multidomain Interventions to improve the COgnitive and fUNctional well-being of old individuals in residential sTructures (I-COUNT) pilot study aimed to evaluate the feasibility and preliminary efficacy of a 6-month multidomain intervention in LTCF residents [10]. The intervention combined structured physical training, cognitive stimulation, a Mediterranean dietary approach enriched with functional foods, and promotion of vaccinations according to the national vaccination plan.

The primary aim of the present analysis was to evaluate the effects of the I-COUNT intervention on physical performance, muscle strength, and sarcopenia prevalence in LTCF residents. Secondary objectives included assessing changes in appendicular skeletal muscle mass (ASMM), body composition, and walking endurance. We hypothesized that the multidomain intervention would improve physical performance and muscle strength, and attenuate the progression of sarcopenia among LTCFs residents compared with usual care.

## 2 Methods

### 2.1 Study design and setting

The I-COUNT study was based on a pilot parallel-group cluster-randomized controlled trial conducted in two LTCFs in Italy: AltaVita-IRA (Padova) and Azienda Speciale Cremona Solidale (Cremona). The study protocol was approved by the Ethics Committees of the participating centers (Cremona: CET 4/24; Padova: CET-ACEV 5982/U6/24), registered on ClinicalTrials.gov (NCT06820710) and then published [10].

Randomization was performed at the cluster level (separate floors or buildings within each LTCF) to minimize the risk of contamination. The SAS Plan procedure was used to randomize each cluster to the multidomain intervention or the usual care group.

Eligible residents were aged ≥70 years, residing in the LTCF for at least six months and with a Mini-Mental State Examination (MMSE) score ≥18. Exclusion criteria included severe disability preventing participation to the training, life expectancy or estimated length of stay in the LTCFs<6 months, previous gastrectomy or colectomy, percutaneous endoscopic gastrostomy or nasogastric tube feeding, dysphagia, history of psychiatric illness, inability to complete psychometric assessments, or inability to walk. The study screening was performed in January 2025, and baseline assessments (T0) in February; the intervention was started in March and was concluded six months later. A comprehensive re-evaluation of all baseline domains was conducted at 6 months in September (T2), after the end of the intervention. Further assessments are envisaged 3 months after the end of the intervention (T3), and will be considered in future analyses (Supplementary Fig. A.1).

### 2.2 Baseline evaluation

• After obtaining informed consent, sociodemographic characteristics, medical and pharmacological history, vaccination records, and Multidimensional Prognostic Index (MPI) [11] were collected. The MPI was derived from Activities of Daily Living (ADL), Instrumental Activities of Daily Living (IADL), Short Portable Mental Status Questionnaire (SPMSQ), Mini Nutritional Assessment-Short Form (MNA-SF), Exton-Smith Scale (ESS), Cumulative Illness Rating Scale (CIRS), and the number of drugs. The MPI was also used to classify participants as robust (≤0.33), pre-frail (0.34–0.66), or frail (>0.66) [12].
• A comprehensive whole-body bioelectrical impedance analysis (BIA) was performed by trained personnel using a tetrapolar configuration, according to standardized procedures, with participants resting in a supine position for at least 5 min before the measurement begins. Appendicular skeletal muscle mass (ASMM) was derived considering the equations by Sergi et al. [13], obtaining then the appendicular skeletal muscle mass index (ASMMI) by dividing ASMM by the subject’s height in meters squared. Anthropometric measurements included body weight, height, calf circumference, and mid-upper arm circumference; when participants could not maintain an upright posture, Chumlea’s equations height was applied based on knee-to-heel length [14]. Body mass index (BMI) was derived as weight (kg) divided by height in meters squared.
• Handgrip strength was measured using DynEx JAMAR TM Plus + electronic dynamometers (Patterson Medical, Green Bay, WI, USA); three measurements per hand were performed, and the final value was calculated as the maximum measure from dominant and non-dominant hand.
• The Short Physical Performance Battery (SPPB) [15] was evaluated to assess balance (side-by-side stands, semi-tandem test, and tandem test), gait speed (4-meter walk), and lower limb strength (5-repetition chair stands test); the final scores ranged between 0 and 12, with higher scores indicating better physical performance.
• Walking endurance was evaluated considering the 6-minute walking test distance. Participants were allowed to use their habitual walking aids (e.g., walker, cane), and their use was recorded. Participants unable to walk independently nor with assistance were excluded from this assessment.
• Sarcopenia was defined considering European Working Group on Sarcopenia in Older People 2 (EWGSOP2) criteria [16].

### 2.3 Multidomain intervention

• Physical training was based on three times/week group sessions (3–5 participants) for six months, with the supervision of a physiotherapist or a motor science expert. Each session was conducted for 40 min, and was based on the VIVIFRAIL multicomponent program [17,18], including [1]: resistance exercises for upper and lower limbs (chair stands, step-ups, arm raises with resistance) [2]; progressive balance training (static and dynamic exercises) [3]; aerobic walking adapted to individual capacity. The intensity of each exercise was personalised based on the baseline assessment and progressively increased.
• Cognitive training was based on two 45-minute sessions each week for six months, in small groups (3–5 participants) with similar cognitive levels, under neuropsychologist supervision. These sessions involved either classical or computerised stimulation based on RECODE platform, a new tool designed by the University of Padua to stimulate the main cognitive domains, including attention, working memory, visual memory, language, orientation and executive functions (available at https://osf.io/eqgyn/?view_only=fa14090e85f7424abf73bf583871b529). Exercises progressed in difficulty based on individual performance (for RECODE, 80% accuracy was considered as threshold for progression).
• In relation to the nutritional component, residents were already following a Mediterranean-style diet as standard meal planning provided by each LTCF. For this reason, the nutritional component of intervention focused on reinforcing adherence to Mediterranean diet, with particular attention to extra-virgin olive oil consumption, and participants took part into structured tasting sessions with experts to enhance their appreciation for sensory properties. In addition, they were asked to consume two specific functional foods: sourdough bread fortified with olive leaf polyphenols [19], and artichokes enriched with Lacticaseibacillus paracasei IMPC2.1 probiotic strain [20].
• A vaccination component, based on recommendations from the national immunization plan for adults aged ≥65 years, was included in the intervention; detailed procedures are described in the study protocol.
• During both the physical and cognitive training sessions, a camera-based remote photoplethysmography (rPPG) app was used to measure heart rate, breath rate, and SpO2 at the beginning and at the end of the session in a sub-sample of the participants. The system leverages subtle color changes in facial skin to derive cardiovascular and respiratory parameters in a completely contactless manner, using the built-in camera of a smartphone as the acquisition device. Heart rate and breath rate were estimated via frequency analysis of the reconstructed rPPG signal, while SpO2 was derived from the ratio of pulsatile and non-pulsatile components of the red and blue color channels. As demonstrated in Caroppo et al. [21], the accuracy of vital signs estimation is about 98%.

Participants in the control group continued usual activities and care, including unstructured recreational and social engagement (e.g. word puzzles, board games, reading), non-structured physical activities, and standard diet. They received general information on healthy lifestyle but no structured physical, cognitive, or nutritional intervention.

### 2.4 Outcomes

The present analyses were based on data collected at baseline (T0) and after completion of the 6-month intervention (T2). In this analysis, we evaluated changes in physical performance (SPPB), muscle strength (handgrip strength), body composition, and sarcopenia prevalence as secondary and exploratory outcomes.

### 2.5 Statistical analysis

The sample size determination has been previously described in the [10]. Briefly, 60 participants per group (120 in total), were planned across the two LTCFs. Although enrolment reached 90 participants in total, this sample size exceeds the number of 30 individuals in the intervention group and 30 individuals in the control group suggested by Sim and Lewis to maximize precision and efficacy for pilot studies [22].

Continuous variables are presented as means ± standard deviation (SD) or median and interquartile range (IQR) for baseline characteristics, while categorical variables are expressed as frequencies and percentages.

In relation to continuous outcomes, repeated-measures analyses were considered using linear mixed-effects models with Restricted Maximum Likelihood (REML) estimation. Fixed effects included group (intervention vs control), time (T0 vs T2), and group × time interaction; age, sex, center, and baseline value of the dependent variables were considered as covariates. A random intercept for each participant was included to account for within-subject correlation for repeated measures. Within-group changes were evaluated using linear contrasts comparing values at T2 with values at the baseline T0, separately for each group. Between-group differences were assessed through the group × time interaction term. Tukey adjustments for multiple comparisons were applied. Given the small number of clusters, the inclusion of a separate random effect for cluster was not feasible, and to account for between-cluster variability, study center was included as a fixed effect. To explore whether baseline nutritional status modified the response to the intervention, an interaction term between MNA total score at baseline and group was added to the linear mixed-effects models.

For the dichotomous outcome sarcopenia, generalized linear mixed-effects models with a binomial distribution and logit link function were used. Fixed effects included group, time, and group × time interaction; age, sex, and center were considered as covariates (baseline value was not included as a covariate due to the binary nature of the outcome). A random intercept for each participant was also included to account for within-subject correlation for repeated measures. Results are presented as predicted prevalence with 95% Confidence Intervals (CI) and as odds ratios (OR) with corresponding 95% CI. Effect sizes were calculated: Cohen’s d was considered for continuous outcomes, while for the dichotomous outcome, they were quantified using the OR.

Linear mixed-effects models with random intercepts for participants and training session and AR [1] residual structure were fitted to evaluate temporal trends in breath rate, heart rate and SPO2 measured through photopletismography through training sessions.

Tests were two-sided, with a significance level *p* < 0.05. Analyses were conducted considering the modified intention-to-treat principle, including all participants who completed at least one follow-up assessment.

Statistical analyses were performed using SAS software, version 9.4 (SAS Institute Inc., Cary, NC, USA).

## 3 Results

### 3.1 Baseline characteristics

**Fig. 1:**
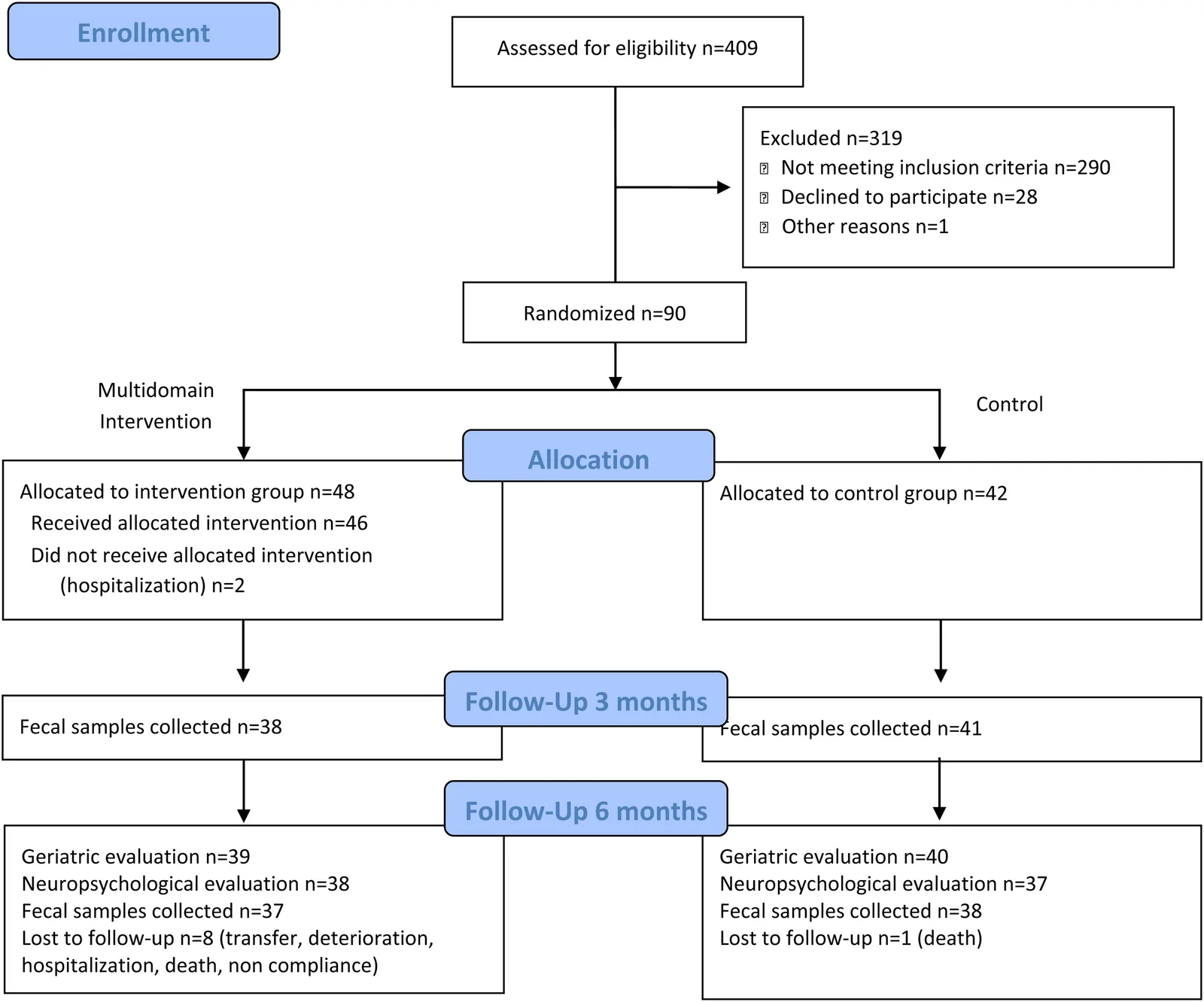
Study flow diagram of participant recruitment, randomization, and follow-up.

A total of 409 residents from the two participating Italian LTCFs were screened, of whom 90 were randomized: 48 to the intervention group and 42 to the control group (Fig. 1). Two participants in the intervention group were unexpectedly hospitalized and transferred immediately after randomization and therefore did not receive the intervention. The remaining participants were followed for six months. Participants were very old (mean age 87.6 ± 5.6 years), predominantly female (71.1%), and presented a high burden of multimorbidity and polypharmacy (81.1%). More than half had an educational level corresponding to elementary school or lower.

Baseline clinical and functional characteristics were well balanced between groups (Table 1). In particular, CIRS comorbidity and severity scores, frailty status according to MPI, BMI classes, and the prevalence of sarcopenia defined according to EWGSOP2 criteria were comparable. No significant differences were observed in physical performance (SPPB), muscle strength (handgrip), cognitive status (MMSE and ACE-R), or depressive symptoms (GDS) (all *p* > 0.05).

**Table 1:** Baseline characteristics of the study participants.

	(*n* = 90)	Intervention Group (*n* = 48)	Control Group (*n* = 42)
Age, years			
Mean ± SD	87.6 ± 5.6	86.8 ± 5.5	88.6 ± 5.6
Range (Min, Max)	73, 100	73, 97	76, 100
Sex, females, *n* (%)	64 (71.1)	36 (75.0)	28 (66.7)
Education, elementary school or less, *n* (%)	49 (54.4)	23 (48.9)	26 (61.9)
CIRS Comorbidity Index, mean ± SD	4.2 ± 2.1	4.2 ± 2.2	4.1 ± 2.1
CIRS Severity Index, mean ± SD	1.9 ± 0.4	1.9 ± 0.4	1.9 ± 0.4
Polypharmacy (5+ drugs), *n* (%)	73 (81.1)	34 (81.0)	39 (81.3)
Frailty according to MPI, *n* (%)			
Robust	14 (15.6)	8 (16.7)	6 (14.3)
Pre-frail	71 (78.9)	39 (81.3)	32 (76.2)
Frail	5 (5.6)	1 (2.1)	4 (9.5)
BMI, *n* (%)			
Underweight	6 (6.7)	2 (4.3)	4 (9.5)
Normal weight	40 (44.9)	23 (48.9)	17 (40.5)
Overweigth	31 (34.8)	14 (29.8)	17 (40.5)
Obesity	12 (13.5)	8 (17.0)	4 (9.5)
Calf circumference, cm, mean ± SD	32.6 ± 3.7	32.7 ± 3.7	32.4 ± 3.7
Arm circumference, cm, mean ± SD	27.8 ± 4.1	27.4 ± 4.2	28.2 ± 4.0
ASMM, kg, mean ± SD	17.8 ± 4.3	17.1 ± 3.9	18.6 ± 4.6
6-min walking test, meters, mean ± SD	198 ± 114	210 ± 122.2	183.4 ± 103.4
SPPB, score, mean ± SD	4.4 ± 2.9	4.3 ± 2.7	4.5 ± 3.1
Handgrip strength, kg			
Females, mean ± SD	13.9 ± 5.0	14.3 ± 5.1	13.5 ± 4.9
Males, mean ± SD	23.1 ± 6.6	25.5 ± 7.4	21.1 ± 5.3
Sarcopenia (EWGSOP2 criteria), *n* (%)	31 (34.4)	18 (37.5)	13 (31.0)
MMSE score (max 30), mean ± SD	24.1 ± 4.0	24.8 ± 3.9	23.4 ± 4.0
ACE-R score (max 100), mean ± SD	65.4 ± 15.6	67.0 ± 16.5	62.4 ± 13.5
GDS 30 score (max 30), mean ± SD	11.4 ± 6.1	12.2 ± 5.9	10.5 ± 6.3

Abbreviations: ACE-R (Addenbrooke’s Cognitive Examination); ASMM (Appendicular Skeletal Muscle Mass); BMI (Body Mass Index); CIRS (Cumulative Illness Rating Scale); EWGSOP2 (European Working Group on Sarcopenia in Older People 2); GDS (Geriatric Depression Scale); MMSE (Mini-Mental State Examination); MPI (Multidimensional Prognostic Index); SPPB (Short Physical Performance Battery).

### 3.2 Retention, adherence, safety and acceptability

Retention and adherence are reported in full in the feasibility manuscript [10]. Briefly, retention during the intervention period was good, and at the six-month follow-up (T2), nine participants (10.0%) withdrew from the study due to clinical deterioration, death, transfer to another facility, or non-adherence to planned activities.

Adherence to the multidomain intervention was generally high, with 78% median adherence to physical training and 80% to cognitive training. Consumption of functional foods was more heterogeneous, with median adherence of 50% for functional bread and 67% for functional artichokes, respectively.

No serious adverse events attributable to the intervention were reported. Vital signs were monitored before and after physical training sessions, and no premature termination for safety concerns was necessary. Participants reported high satisfaction with the physical training component. Using a 5-point Likert scale, median ratings were 5 for simplicity, comfort, and willingness to continue the program. Exercises were perceived as easy to follow and well tolerated throughout the intervention (median ratings 5).

No significant increase during sessions was observed for heart rate (β = 0.06 bpm/min, *p* = 0.741), respiratory rate (β = 0.06 breaths/min, *p* = 0.335), or SPO2 (β = −0.01 %/min, *p* = 0.937).

### 3.3 Changes in physical performance and muscle strength

**Fig. 2:**
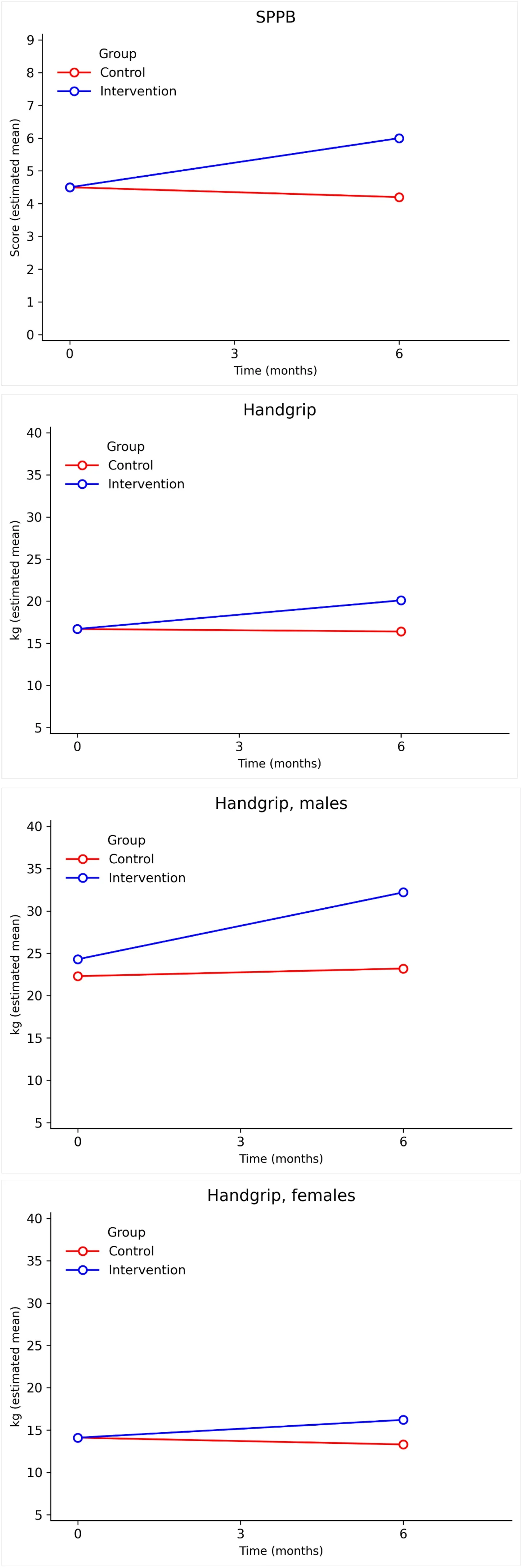
Estimated variation in relation to physical performance measures for the I-COUNT study (T2 vs T0).

The intervention led to a meaningful improvement in physical performance and muscle strength (Table 2; Fig. 2). At six months, SPPB increased by +1.6 points in the intervention group, while remaining essentially unchanged in controls (+0.3). The between-group difference was significant (+1.8 points, *p* = 0.001) with a moderate-to-large effect size (*d* = 0.64). Muscle strength also improved in the intervention group. Mean handgrip strength increased by +3.2 kg, whereas it remained stable in controls (−0.1 kg), resulting in a significant between-group difference (+3.8 kg, *p* = 0.026; *d* = 0.54). Sex-stratified analyses suggested a stronger response among males. In this subgroup, handgrip increased markedly in the intervention group (+8.3 kg) while remaining stable in controls, producing a large effect size (*d* = 1.60). Among females, the increase in the intervention group was smaller (+2.0 kg) and did not reach statistical significance. Finally, changes in the 6-minute walk test were modest and did not differ significantly between groups. The estimated change was +11.7 m in the intervention group and +3.5 m in controls, with no significant between-group difference (*p* = 0.904). It should be noted that the 6-minute walk test was performed by 84 participants (88%) at baseline, including those using walking aids.

**Table 2:** Mixed models related to anthropometric and physical performances measures in the I-COUNT study (T2 vsT0; mixed models adjusted for age, sex, and values at T0).

	Mean differences *within-group*								Mean differences *between-group* at T2		
	Intervention group				Control group						
	T0	T2	Δ T2–T0	p-value	T0	T2	Δ T2-T0	p-value	Δ	p-value^a^	Effect size
	Mean (SE)	Mean (SE)	Mean (SE)		Mean (SE)	Mean (SE)	Mean (SE)		Mean (SE)		d
BMI, kg/m^2^	25.2 (0.2)	25.0 (0.2)	−0.2 (0.2)	0.781	25.2 (0.2)	25.6 (0.2)	0.4 (0.3)	0.449	−0.6 (0.3)	0.087	0.01 (−0.42, 0.42)
Calf circumference, cm	32.5 (0.5)	32.5 (0.5)	0.3 (0.7)	0.979	32.4 (0.5)	31.9 (0.5)	−0.4 (0.7)	0.935	0.3 (0.8)	0.890	−0.01 (0−0.42, 0.41)
Mid-arm circumference, cm	27.8 (0.3)	28.0 (0.3)	0.2 (0.4)	0.969	27.9 (0.3)	27.7 (0.3)	−0.3 (0.5)	0.925	0.3 (0.5)	0.458	0.05 (−0.37, 0.47)
ASMM, kg	17.7 (0.3)	17.8 (0.4)	−0.1 (0.5)	0.991	18.0 (0.3)	16.9 (0.3)	−1.1 (0.5)	0.072	−0.9 (0.5)	0.057	−0.32 (−0.11, 0.75)
6-min walking test, m	199.7 (8.1)	211.4 (9.4)	11.7 (12.5)	0.776	198.5 (8.9)	202.0 (9.8)	3.5 (13.1)	0.993	9.3 (13.7)	0.904	0.11 (−0.33, 0.54)
SPPB, score	4.4 (0.3)	6.0 (0.3)	1.6 (0.4)	**<0.001**	4.5 (0.3)	4.2 (0.3)	0.3 (0.4)	0.894	1.8 (0.4)	**0.001**	0.64 (0.21, 1.07)
Handgrip strength, kg	16.8 (0.7)	20.0 (0.8)	3.2 (1.0)	**0.009**	16.3 (0.7)	16.2 (0.8)	−0.1 (1.1)	0.999	3.8 (1.1)	**0.026**	0.54 (0.12, 0.97)
Females	14.1 (0.7)	16.2 (0.8)	2.0 (1.0)	0.189	14.1 (0.8)	13.3 (0.9)	−0.8 (1.1)	0.906	2.9 (1.2)	0.067	0.52 (0.02, 1.03)
Males	24.2 (1.8)	32.5 (2.2)	8.3 (2.7)	**0.022**	22.2 (1.6)	23.2 (1.9)	0.9 (2.4)	0.979	9.3 (3.1)	**0.023**	1.60 (0.72, 2.49)

Abbreviations: ASMM (Appendicular Skeletal Muskle Mass); BMI (Body Mass Index); SE (Standard Error); SPPB (Short Physical Performance Battery).

In **bold** are reported p-values <0.05.

a p-value for the interaction group × time.

To assess whether baseline nutritional status modified the response to the intervention, the MNA baseline score was included in the models for muscle strength and physical performance. A borderline significant interaction between MNA baseline score and group was observed for handgrip strength (*p* = 0.061), suggesting that participants with better baseline nutritional status may have had a greater improvement in handgrip strength. However, the estimated between-group difference at six months after adjustment for baseline MNA remained of similar magnitude (+3.6 kg, *p* = 0.010), indicating that the intervention effect on handgrip was not explained by differences in baseline nutritional status. No significant interaction between MNA baseline score and group was observed for the 6-minute walk test (*p* = 0.167) and for SPPB (*p* = 0.369), suggesting that the effect of the intervention on physical performance was not modified by baseline nutritional status.

### 3.4 Changes in body composition

Changes in body composition were modest overall (Table 2). BMI slightly decreased in the intervention group and slightly increased in controls, with a borderline between-group difference (*p* = 0.087) but minimal effect size. Similarly, no significant changes were observed in calf or mid-upper arm circumference. Estimated ASMM remained stable in the intervention group but tended to decline in controls. The between-group difference at six months approached statistical significance (+0.9 kg, *p* = 0.057), suggesting a potential protective effect of the intervention on muscle mass.

### 3.5 Sarcopenia prevalence

At baseline, sarcopenia prevalence according to EWGSOP2 was similar between groups (37.5% vs 31.0%, *p* = 0.514, Table 1). Adjusted prevalence estimates accounting for age, sex, and center confirmed this comparability (Table 3: intervention group 37.1%, 95% CI 22.2–54.9%; control group 28.4%, 95% CI 14.9–47.3%). After six months, the estimated prevalence of sarcopenia decreased in the intervention group (15.4%, 95% CI 6.4–32.6%), whereas it remained stable in the control group (29.9%, 95% CI 14.8–51.2%). Within-group analysis showed stable odds of sarcopenia in the intervention group (OR = 0.93, 95% CI 0.32–2.70, *p* = 0.890), but a significant increase in the control group (OR = 3.24, 95% CI 1.08–9.75, *p* = 0.036). The group × time interaction showed borderline statistical significance (*p* = 0.109) (Table 3). No significant differences were detected between males and females.

**Table 3:** Prevalence and estimated variation related to sarcopenia in the I-COUNT study (T2 vs T0, generalized linear models adjusted for sex and age).

	*Within-group*								*Between-group* at T2	
	Intervention group				Control group					
	T0	T2	OR sarcopenia (95% CI)	p-value	T0	T2	OR sarcopenia (95% CI)	p-value	OR sarcopenia T2	p-value^a^
	Prevalence (95% CI)	Prevalence (95% CI)			Prevalence (95% CI)	Prevalence (95% CI)			Intervention vs control group	
Sarcopenia	37.1 (22.2, 54.9)	15.4 (6.4, 32.6)	0.93 (0.32, 2.70)	0.890	28.4 (14.9, 47.3)	29.9 (14.8, 51.2)	3.24 (1.08, 9.75)	**0.036**	0.67 (0.22, 2.02)	0.109

Abbreviations: CI (Confidence Interval); OR (Odds Ratio).

In **bold** are reported p-values <0.05.

a p-value for the interation group × time.

## 4 Discussion

This pilot cluster-randomized trial addresses a critical gap in the literature by providing real-world evidence on the implementation of a multidomain intervention in LTCF residents, a population that is typically excluded from or underrepresented in intervention trials. While feasibility, adherence and acceptability have been presented in a dedicated manuscript [10], the present paper is related to functional outcomes. In particular, potential improvements in physical performance and muscle strength were observed, with a tendency toward preservation of muscle mass and a more favorable trajectory of sarcopenia in the intervention group compared to usual care group. Importantly, these results should be interpreted in light of the pilot nature of the I-COUNT study and the exploratory nature of the outcomes. As physical performance, muscle strength, body composition, and sarcopenia were pre-specified as secondary and exploratory outcomes, these findings should be interpreted as hypothesis-generating rather than confirmatory

In this context, the intervention was also shown to be safe and well tolerated, with high adherence to supervised components and no intervention-related adverse events. Physiological parameters remained stable during training sessions, supporting the applicability of such programs even in very old and clinically complex individuals. These findings extend previous evidence on multicomponent and multidomain interventions, which have demonstrated feasibility and efficacy mainly in community-dwelling populations, but remain less explored in institutionalized settings [17,18]

It is not surprising that the most consistent effects were observed for SPPB, a composite and sensitive measure of lower-limb function that captures multiple domains targeted by the intervention. Our findings are in line with previous studies showing that multicomponent physical training programs, particularly those based on the VIVIFRAIL model, are associated with significant improvements in physical performance and muscle strength in older adults [18]. Similarly, multidomain interventions combining physical training, cognitive training, and nutritional components have demonstrated beneficial effects on intrinsic capacity, especially in locomotion and vitality domains [23]. Evidence from both randomized trials and narrative syntheses suggests that improvements in SPPB are more likely when programs include structured and supervised exercise, rather than simple lifestyle advice, particularly when programs integrate resistance and balance training [24]. In this context, the multidomain and multicomponent nature of our intervention, together with the good adherence observed, potentially played a major role in driving the observed effects in physical performance. From a mechanistic perspective, resistance and balance training are known to improve neuromuscular function, muscle strength, and postural control [[24], [25], [26]], which are key determinants of SPPB performance. At the same time, the multidomain nature of the intervention including cognitive stimulation and nutritional support, with reinforcement of a Mediterranean-style diet and the introduction of functional foods, may have contributed by enhancing overall functional reserve and engagement, thereby facilitating a more effective response to physical training, with potential benefits on muscle metabolism and low-grade inflammation.

In contrast, no significant effect was observed for the 6-minute walk test. This finding may reflect the different physiological constructs captured by the two measures: while SPPB primarily reflects lower-limb strength, balance, and short-distance mobility, the 6-minute walk test is more strongly influenced by aerobic capacity, cardiopulmonary reserve, and overall endurance. These components may be less responsive to the type, intensity, and duration of exercise implemented in our study, particularly in very old and clinically complex individuals. Moreover, the 6-minute walk test may be less sensitive to short-term functional changes in this population, where improvements are more likely to occur in basic functional tasks rather than in sustained walking performance. This pattern is consistent with the type and intensity of the intervention delivered, which primarily targeted strength and balance rather than aerobic capacity.

Consistent with previous literature [18,23], the intervention also resulted in a significant improvement in muscle strength, as reflected by handgrip. Notably, the effect was more pronounced among males. This sex-specific pattern may be partly explained by higher baseline muscle mass and greater absolute strength in men, which may allow for a larger margin of improvement in response to resistance-based training, as suggested by previous evidence on sex-related differences in exercise responsiveness in older adults [27]. In contrast, in very old women—who typically present lower muscle reserves and a higher prevalence of dynapenia [28], the same intervention may result in smaller and more variable gains. However, these subgroup findings should be interpreted with caution due to the pilot nature of the study, the limited sample size and exploratory nature of these analyses.

We also explored whether baseline nutritional status influenced the response to the intervention. Although a borderline interaction between MNA score and group was observed for handgrip strength, suggesting a potential greater improvement among participants with better nutritional status, the overall effect of the intervention remained significant after adjustment. This suggests that, while adequate nutritional status may enhance responsiveness to exercise [29,30], the observed improvement in muscle strength was not solely driven by baseline nutritional differences. No such interaction was observed for SPPB or walking endurance, suggesting that the effect of the intervention on physical performance was relatively consistent across different nutritional strata, while muscle strength may be more sensitive to baseline biological reserves.

Finally, no significant changes were observed in anthropometric measures, including BMI and peripheral circumferences. This finding is consistent with previous studies in older and frail populations, where improvements in physical performance and muscle strength are not necessarily paralleled by short-term changes in body composition [31,32]. In particular, adaptations to exercise in very old individuals often occur initially at the neuromuscular level [33], with gains in strength and function preceding detectable changes in muscle mass [34]. In this context, the lack of significant variation in anthropometric parameters may also reflect the relatively short duration of the intervention, as well as the limited sensitivity of these measures to capture subtle changes in body composition. Notably, appendicular skeletal muscle mass tended to be preserved in the intervention group while declining in controls, suggesting a potential protective effect. This pattern is consistent with the observed divergence in sarcopenia prevalence between groups, which may be interpreted as the combined effect of improved muscle strength in the intervention group and a tendency toward muscle mass decline in controls. Although ASMM was slightly lower in the intervention group at baseline, models were adjusted for baseline values, and the magnitude of the difference is unlikely to fully explain the decline observed in the control group through regression to the mean.

Overall, these findings suggest that multidomain interventions in LTCFs may primarily act through improvements in muscle strength and preserving muscle mass, with potential effects on the trajectory of sarcopenia, even in the absence of overt anthropometric changes. These results should be considered as preliminary and warrant confirmation in larger trials.

### 4.1 Limitations and strengths

This study has several limitations. The relatively small sample size and pilot design may have limited the power to detect significant differences for certain outcomes, particularly body composition measures. The inclusion of only two LTCFs may limit the generalizability of the results. In addition, the 6-month duration may have been insufficient to observe more pronounced changes in anthropometric parameters and muscle mass. Another limitation is related to the partial implementation of the multidomain intervention originally planned. The vaccination component could not be effectively delivered during the study period, considering that the intervention took place between March and August 2025, outside the seasonal window for influenza and COVID-19 vaccinations. A review of vaccination records in the five years preceding baseline showed that a proportion of participants (38% in the overall sample) had already received pneumococcal vaccination in the five years preceding baseline, and none had received herpes zoster or diphtheria-tetanus-pertussis vaccination, suggesting that opportunities for vaccination may have been missed. Future trials should systematically assess at baseline vaccination history and establish pathways with referring physicians to address individual vaccination needs. Finally, the multidomain nature of the intervention did not allow disentangling the specific contribution of each component, and the role of the nutritional intervention remains exploratory.

Despite these limitations, the study has important strengths. The cluster-randomized design enabled implementation in a real-world long-term care setting, reducing contamination between groups. The inclusion of very old and clinically complex individuals enhances the clinical relevance of the findings. Moreover, the multidomain approach reflects current geriatric care models and allowed the evaluation of integrated effects across multiple functional domains.

## 5 Conclusions

This pilot cluster-randomized trial shows that a multidomain intervention is feasible and safe in LTCF residents, and may improve physical performance and muscle strength with a potential impact on sarcopenia progression. Given the pilot nature of the study, these results should be interpreted with caution, and larger randomized trials are needed to confirm these findings. Overall, these findings support further investigation of integrated, multidomain interventions to promote functional health in very old and frail populations.

## CRediT authorship contribution statement

Author contributions: CC: Writing - original draft, Writing - review & editing, Data curation, Conceptualization, Formal analysis; GP: Writing - original draft, Data curation; MVP: Writing - review & editing, Data curation; GSc: Writing - original draft, Data curation; AB: Writing - review & editing, Data curation; EM: Writing - original draft, Writing - review & editing, Data curation; AG: Writing - review & editing, Data curation; ZR: Writing - original draft, Writing - review & editing, Data curation, Conceptualization; GSe: Writing - review & editing, Supervision; MDR: Writing - review & editing; Supervision; SG: Project administration, Supervision; SM: Writing - review & editing; Conceptualization, Supervision, Project administration, Funding acquisition; AM: Writing - review & editing, Conceptualization, Supervision, Project administration; MN: Writing - original draft, Writing - review & editing, Formal analysis, Conceptualization, Supervision, Project administration, Funding acquisition. All authors have read and agreed to the final version of the manuscript.

## Declaration of Generative AI and AI-assisted technologies in the writing process

The authors confirm that no generative AI or AI-assisted technologies were used in the scientific writing process, data analysis, or the creation/alteration of figures, images, and artwork for this manuscript.

## Funding

This work was supported by the Next Generation EU project [DM 1557 October 11, 2022], National Recovery Resilience Plan, Investment PE8 – Project Age-It: “Ageing Well in an Ageing Society”. The views and opinions expressed are only those of the authors and do not necessarily reflect those of the European Union or the European Commission. Neither the European Union nor the European Commission can be held responsible for them.

## Data availability statement

The datasets analyzed for the current study are not publicly available due to privacy and ethical restrictions.

## Declaration of competing interest

The authors declare that they have no known competing financial interests or personal relationships that could have appeared to influence the work reported in this paper.
