# Corrigendum to “Effects of a Multidomain Intervention on Physical Performance and Sarcopenia in Long-Term Care Facility Residents: the I-COUNT Pilot Randomized Controlled Trial” [J Nutr Health Aging, 30 (2026) 100943]

**DOI:** 10.1016/j.jnha.2026.100970

**Published:** 2026-09-04

**Authors:** Chiara Ceolin, Giulia Pasolini, Mario Virgilio Papa, Giacomo Scotti, Alvise Bobbo, Eleonora Macchia, Alessia Ghidini, Zaira Romeo, Giuseppe Sergi, Marina De Rui, Simona Gentile, Stefania Maggi, Alessandro Morandi, Marianna Noale, for the ICOUNT study group

**Affiliations:** a Department of Medicine (DIMED), University of Padua, Padua, Italy; b Department of Neurobiology, Care Sciences and Society, Karolinska Institutet and Stockholm University, Aging Research Center, Stockholm, Sweden; c Department of Clinical and Experimental Science, University of Brescia, Italy; d Neuroscience Institute, National Research Council, Padova, Italy; e Azienda Speciale Cremona Solidale, Cremona, Italy; f Geriatrics Unit, University Hospital of Padua, Padua, Italy

After publication, the authors noticed an error in Alessandro Morandi’s affiliations.

Alessandro Morandi is affiliated with:

- Department of Clinical and Experimental Science, University of Brescia, Italy;
- Azienda Speciale Cremona Solidale, Cremona, Italy.

The authors apologize for this error and for any inconvenience it may have caused.

