# Supplementary material for: Effects of a multidomain intervention on physical performance and sarcopenia in long-term care facility residents: The I-COUNT pilot randomized controlled trial
Source: J Nutr Health Aging. 2026 Aug 13;30(10):100943. doi: 10.1016/j.jnha.2026.100943 (PMC13495356; doi:10.1016/j.jnha.2026.100943)
Supplement: Supplementary file 1 [file mmc1.pdf]

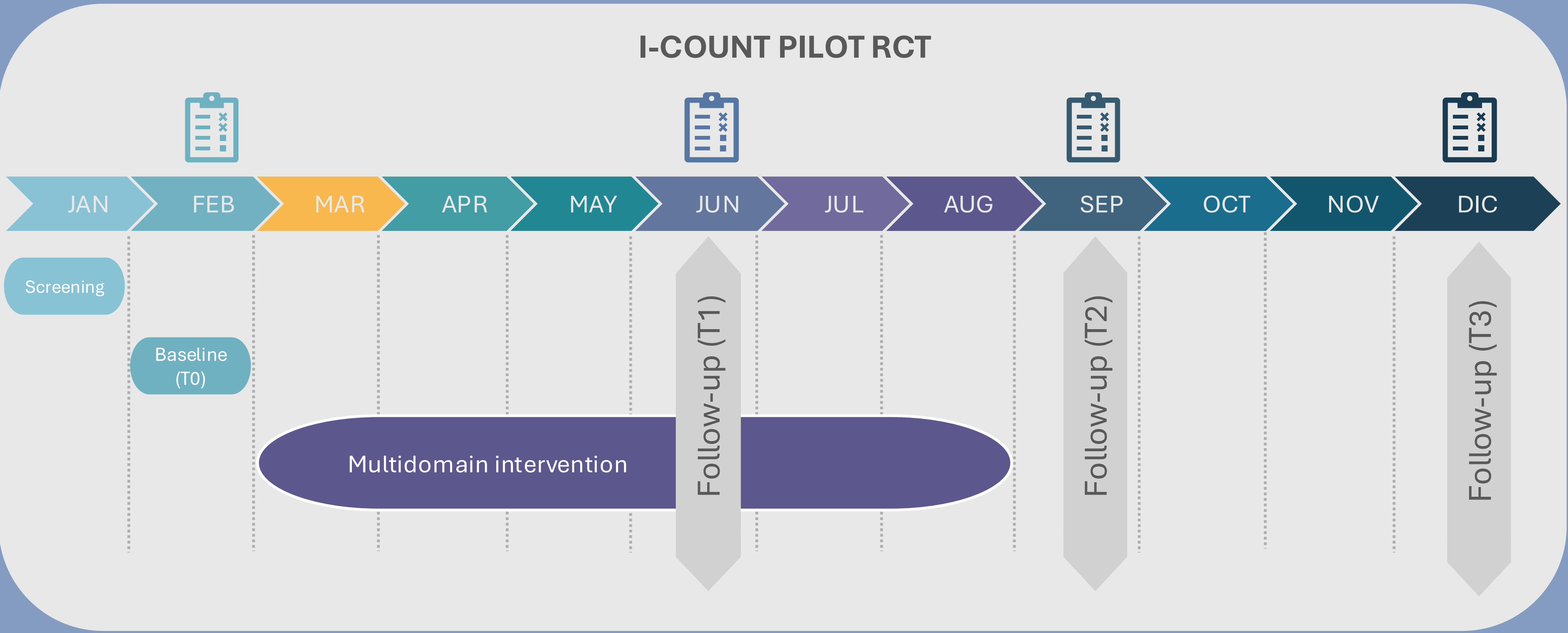

I-COUNT PILOT RCT
JAN
FEB
MAR
APR
MAY
JUN
JUL
AUG
SEP
OCT
NOV
DIC
Screening
Baseline (T0)
Multidomain intervention
Follow-up (T1)
Follow-up (T2)
Follow-up (T3)
